# Role of Bispecific Antibodies in Relapsed/Refractory Diffuse Large B-Cell Lymphoma in the CART Era

**DOI:** 10.3389/fimmu.2022.909008

**Published:** 2022-07-19

**Authors:** Eva González Barca

**Affiliations:** Hematology Department, Catalan Institute of Oncology, University of Barcelona, IDIBELL, Barcelona, Spain

**Keywords:** diffuse large B-cell lymphoma, relapsed/refractory, bispecific antibodies, non-Hodgkin lymphoma, post CART therapy

## Abstract

Diffuse large B-cell lymphoma is an aggressive and biologically heterogeneous disease. R-CHOP is the standard first line therapy and cures more than 60% of patients. Salvage high-dose chemotherapy with autologous stem cell transplant remains the standard second-line treatment for relapsed or refractory patients, and recently, three CD19 chimeric antigen receptor T cells (CART) cell products have been approved beyond 2 prior lines of systemic therapy. Nevertheless, some patients are not eligible for transplant or CARTs, or progress after these treatments. In this context, IgG-like bispecific antibodies (BsAbs) have been designed to treat B‐cell lymphomas. They combine two different monospecific antigen‐binding regions that target CD20 on B cells and engage T cells *via* CD3 in a 1:1 or 2:1 CD20:CD3 antigen binding fragment (Fab) format. The results of different phase 1 trials with BsAbs, including mosunetuzumab, glofitamab, epcoritamab and odeonextamab, have been recently published. They are infused intravenously or subcutaneously, and have a favorable toxicity profile, with reduced cytokine release syndrome and neurological toxicity. Moreover, these BsAbs have demonstrated very promising efficacy in B-cell lymphomas, including in aggressive lymphomas. New trials are currently ongoing to confirm BsAbs efficacy and tolerability, as well as to explore its efficacy in different lines of therapy or in combination with other drugs.

## Introduction

Diffuse large B-cell lymphoma (DLBCL), the most common subtype of non-Hodgkin lymphoma, is an aggressive and heterogeneous disease. Since the late 1990s, six to eight cycles of rituximab, cyclophosphamide, doxorubicin, vincristine, and prednisone (R-CHOP) has been the standard of care (1). More than 60% of patients are cured with this regimen. There have been different trials trying to improve the results of R-CHOP without success, as those in which targeted therapies are added to the R-CHOP backbone: bortezomib (REMoDL-B trial) (2), ibrutinib (PHOENIX trial) (3), or lenalidomide (ROBUST trial) (4), or the trial in which rituximab is replaced by obinutuzumab (a glycoengineered, type II anti-CD20 monoclonal antibody, GOYA trial) (5). Nevertheless, in a recently published phase 3 trial, a modified regimen of R-CHOP (pola-R-CHP), in which vincristine was replaced by polatuzumab vedotin (anti-CD79b antibody-drug conjugate), was compared with the standard R-CHOP, in patients with previously untreated intermediate-risk or high-risk DLBCL, and progression-free survival (PFS) was significantly higher in the pola-R-CHP group than in the R-CHOP group (76.7% vs. 70.2% at 2 years, hazard ratio 0.73), with a similar safety profile in the two groups, although overall survival did not differ significantly (6).

Salvage high-dose chemotherapy with autologous stem cell transplant (ASCT) remains the standard second-line treatment for relapsed or refractory (R/R) patients. However, few patients are cured with this intensive approach, and applicability is limited by comorbidities and advanced age (7). Moreover, patients with refractory disease or relapse within 12 months of ASCT have poor outcomes even with this intense strategies, as it is shown in the SCHOLAR-1 multicenter retrospective study, in which the objective response rate (ORR) to the next line of therapy in such patients was 26% (CR, 7%), with a median overall survival (OS) rate of 6.3 months (8).

Recent novel immunotherapy approaches are changing the treatment landscape for these patients. CD19 chimeric antigen receptor T cells (CARTs), are autologous T cells that have been genetically reengineered using viral transduction to express an anti-CD19 single- chain variable fragment for antigen recognition. Three CD19 CART products have been approved by the US Food and Drug Administration (FDA) and the European Medicines Agency (EMA) [axicabtagene ciloleucel (axi-cel), tisagenlecleucel (tisa-cel), and lisocabtagene maraleucel (liso-cel)], for the treatment of R/R aggressive B-cell lymphomas, including DLBCL, high-grade B-cell lymphoma, transformed follicular lymphoma, and primary mediastinal B-cell lymphoma, after ≥ 2 prior lines of systemic therapy, and they show high response rates with durable remissions (9–11). The most up-to-date data with axi-cel demonstrates an OS rate at 4 years of 44% (12).

Due to these impressive results beyond two lines of therapy, several trials tested CART therapy in second line in high risk DLBCL patients. Three randomized phase 3 clinical trials compared the second line treatment with high-dose chemotherapy followed by ASCT (standard arm), with CART therapy (experimental arm), in high-risk patients with DLBCL, refractory or in early relapse (during the first year after finishing the first line treatment) (13–15). An improvement in event-free survival compared with ASCT was demonstrated in 2 of them (13, 15). As a result of these trials, FDA has approved on April 1^st^, 2022, the use of axi-cel in second line for adult patients with DLBCL refractory or relapsed within 12 months after first-line chemoimmunotherapy. Therefore, CARTs have changed the treatment paradigm for R/R aggressive B-cell lymphomas, although significant toxicities are associated with this therapy, such as cytokine release syndrome (CRS) and immune effector cell-associated neurologic syndrome (ICANS).

Nevertheless, despite the high efficacy of CART therapy, many patients do not respond or relapse, representing a new unmet clinical need. Results from retrospective studies show a median survival for these patients of around 6 months (16–18). There are no standard treatment options. Check points inhibitors have been used in early relapses, resulting In ORR of 40%, as well as lenalidomide based regimens, chemotherapy and radiotherapy (17). Loncastuximab tesirine, a humanized anti-CD19 antibody drug conjugated, has been used in the LOTIS 2 trial, in 13 patients after CART failure, with an ORR of 46% (19). For those patients who achieve a response, a consolidation with allogeneic transplantation should be considered (17).

Other new targeted approaches for R/R DLBCL have been recently approved, as the combination of tafasitamab (anti-CD19 monoclonal antibody) and lenalidomide (20), the combination of polatuzumab vedotin with bendamustine and rituximab (21), and selinexor, an oral inhibitor of exportin 1 (approved by the FDA but not by the EMA) (22). Each of these options should be considered for patients who are poor candidates for ASCT or for CARTs.

The observations that CART cells are capable of achieving very durable remissions supported the T cell‐mediated approach to therapy. With this in mind, a new type of monoclonal antibodies called bispecific antibodies (BsAbs) has been designed. BsAbs combine two different monospecific antigen‐binding regions from different antibodies to achieve a single antibody‐derived molecule with bispecific antigen binding. Currently, there are under development BsAbs that target CD20 on B cells and engage CD3 on T cells in a 1:1 or 2:1 CD20:CD3 antigen binding fragment (Fab) format, to treat B‐cell lymphomas (**Table 1**). There are different types of BsAbs, those that are IgG-like are large, and contain the fragment crystallizable (Fc) region linking the two antibody binding domains, which is an advantage as they have pharmacokinetic profiles that allow intermittent dosing. Thus, BsAbs have emerged as a novel class of off-the-shelf immunotherapies with clear efficacy in R/R aggressive B-cell lymphomas, including for those patients relapsing after CART therapy. This review describes updated data on the toxicity and efficacy of the IgG-like BsAbs that are currently being studied in lymphomas, especially in DLBCL.

**Table 1:** Structure of IgG-like bi‐specific antibodies.

Bi-specific Antibody	Targets	Administration	Structure
**Mosunetuzumab**	CD20 x CD3	IV or SC Step-up doses on C1 (D1, D8, D15) Subsequent 21-day cycles for 8 cycles for patients in CR and up to 17 cycles for those with PR or SD	Humanized mouse IgG1-based heterodimeric antibody
**Glofitamab**	(CD20)_2_ x CD3	IV 21-day cycles up to 12 cycles Seven days before 1,000 mg obinutuzumab	Humanized mouse IgG1-based antibody. Bivalent CD20 binding
**Epcoritamab**	CD20 x CD3	SC Weekly dosing in C1-C2 (D1,D8, D15, D22); every 2 weeks in C3–C6 (D1, D15), every 4 weeks from C7 onward Until disease progression or unacceptable toxicity	Humanized mouse heterodimeric IgG1-based heterodimeric antibody
**Odronextamab**	CD20 x CD3	IV Step-up doses on C1 (D1, D2, D8, D9, D15, D16) Weekly dosing C2–C4 (D1,D8, D15), in 21-day cycles After C4, maintenance treatment every 2 weeks Until disease progression or unacceptable toxicity	Fully human IgG4-based heterodimeric antibody

Ig, immunoglobulin; SC, subcutaneous; IV, intravenous; C,cycle; D, day.

## IgG-Like Bispecific Antibodies

Clinical data available of the four BsAbs that are in clinical development for DLBCL will be discussed, as well as the clinical trials that are ongoing.

### Mosunetuzumab (BTCT4465A)

Mosunetuzumab is a fully humanized IgG1 BsAb against CD20 and CD3 and has been developed in both an intravenous (IV) and subcutaneous (SC) formulation.

The results of the first-in-human trial have been recently published (23). This trial evaluated the safety and efficacy of mosunetuzumab in patients with R/R B-NHL and established the recommended phase II dose. Single-agent mosunetuzumab was administered IV in 3-week cycles, at full dose in cycle 1 day 1 (group A) or with ascending (step-up) doses during cycle 1 on days 1, 8, and 15 (group B), to reduce CRS, for eight or 17 cycles on the basis of tumor response. Two hundred thirty patients were enrolled, 129 with aggressive B-NHL, with a median of 3 previous lines of therapy, 82% of whom were refractory to last therapy. Fifteen patients were treated after failure to CART therapy. Common adverse events of the whole group were neutropenia (28.4%), CRS (27.4%), hypophosphatemia (23.4%), fatigue (22.8%), and diarrhea (21.8%). CRS was mostly low-grade (grade ≥ 3: 1.0%) and mainly confined to cycle 1. In group B, most neurologic adverse events were grade 1-2: headache (17.8%), insomnia (11.2%), and dizziness (10.2%). Grade 3 occurred in 4.1% of patients; however, only two (1.0%) were considered treatment-related; there were no grade 4 or 5 neurologic events. For patients with aggressive B-NHL, ORR was 34.9%, complete remission (CR) was 19.4%, and median duration of response (DoR) was 22.8 months. In patients who were refractory to prior CART therapy (15 with aggressive NHL and 4 with indolent NHL), ORR was 36.8%, and CR rate was 26.3%. The authors conclude that mosunetuzumab has a manageable safety profile and induces durable complete responses, and select the dose of 1/2/60/60/30 mg for the expansion stage of the study. Preliminary results of the SC formulation have been presented, with similar response rates (24).

These promising results have led to different new studies that are showed in **Table 2**. One trial has been design to evaluate mosunetuzumab after failure to CART therapy, others in R/R patients in combination with other drugs as lenalidomide, atezolizumab, polatuzumab, or GemOx (gemcitabine and oxaliplatin); and some trials are designed in the upfront setting combined with CHOP. Some preliminary results from some of these ongoing trials, that confirm the efficacy of mosunetuzumab in different settings with a good safety profile, have been already reported (25–28).

**Table 2:** Ongoing trials with IgG-like bispecific antibodies in lymphoma, with the clinical trial identifier.

**Mosunetuzumab**					
**R/R** **post CART**	**Aggressive NHL**	Mosunetuzumab or glofitamab	R/R DLBCL or transformed FL	Phase 2	NCT04889716
**R/R** **in combination**	**Aggressive NHL**	Mosunetuzumab or glofitamab + **GemOx**	R/R DLBCL or high grade DLBCL	Phase 1b	NCT04313608
	**Indolent NHL**	Mosunetuzumab + lenalidomide vs glofitamab + **lenalidomide**± obinutuzumab	R/R FL	Phase 1/2	NCT04246086
		Mosunetuzumab + **lenalidomide** vs R-lenalidomide	R/R FL	Phase 3	NCT04712097
	**NHL**	Mosunetuzumab IV± **atezolizumab**/Mosunetuzumab SC	R/R NHL and CLL	Phase 1/2	NCT02500407
		Mosunetuzumab (IV or SC) + **polatuzumab vedotin**	R/R NHL (FL, DLBCL, MCL)	Phase 1b/2	NCT03671018
		Mosunetuzumab or glofitamab in combination with **cc-220 and cc-99282**	R/R NHL	Phase 1b	NCT05169515
**First line**	**Aggressive NHL**	Mosunetuzumab + CHOP or polatuzumab vedotin- CHP	Untreated NHL	Phase 1b/2	NCT03677141
		Mosunetuzumab (IV or SC)	Consolidation after frontline immunochemotherapy, or untreated DLBCL (elderly/unfit)	Phase 1/2	NCT03677154
	**Indolent NHL**	Mosunetuzumab (SC) + lenalidomide	FL and MZL	Phase 2	NCT04792502
**Glofitamab**					
**R/R** **post-CART**	**Aggressive NHL**	Mosunetuzumab or glofitamab	R/R DLBCL or transformed FL	Phase 2	NCT04889716
	**NHL**	Glofitamab	R/R NHL	Phase 2	NCT04703686
**R/R** **in combination**	**Indolent NHL**	Mosunetuzumab + lenalidomide vs glofitamab + **lenalidomide**± obinutuzumab	R/R FL	Phase 1/2	NCT04246086
	**Aggressive NHL**	Mosunetuzumab **+ GemOx** or glofitamab + **GemOx**	R/R DLBCL or high grade DLBCL	Phase 1b	NCT04313608
		Glofitamab + **GemOx** vs R-GemOx	R/R DLBCL	Phase 3	NCT04408638
	**NHL**	Glofitamab ± **obinuzutumab**	R/R NHL	Phase 1/2	NCT03075696
		Glofitamab + **atezolizumab** or **polatuzumab vedotin**	R/R NHL	Phase 1b	NCT03533283
		Glofitamab + **RO7227166**	R/R NHL	Phase 1	NCT04077723
		Mosunetuzumab or glofitamab in combination with **CC-220 and CC-99282**	R/R NHL	Phase 1b	NCT05169515
**First line**	**Aggressive NHL**	Glofitamab + R-CHOP or Polatuzumab vedotin- R-CHP	Untreated DLBCL (young, high risk)	Phase 1/2	NCT04914741
		Glofitamab + R-CHOP or Glofitamab + obinu-CHOP	Untreated DLBCL (R/R NHL)	Phase 1b	NCT03467373
**Epcoritamab**					
**R/R** **or first line** **in combination**	**NHL**	Epcoritamab + R-DHAX/C Epcoritamab + GemOx Epcoritamab + R-Lenalidomide Epcoritamab + R-CHOP Epcoritamab + R-B	R/R DLBCL R/R DLBCL R/R FL Untreated DLBCL Untreated FL	Phase 1b/2	NCT04663347
**R/R** **in combination**	**Aggressive NHL**	Epcoritamab vs R-GemOx or R-B	R/R DLBCL	Phase 3	NCT04628494
**Odronextamab**					
**R/R** **monotherapy**	**NHL**	Odronextamab	R/R NHL	Phase 1	NCT02290951
**R/R** **monotherapy**	**NHL**	Odronextamab	R/R NHL	Phase 2	NCT03888105
**R/R in combination**	**NHL**	Odronextamab + cepilimab	R/R NHL	Phase 1	NCT02651662

Ig, immunoglobulin; R/R, relapsed/refractory; NHL, non-Hodgkin lymphoma; MCL, mantle cell lymphoma; MZL, marginal zone lymphoma; FL, follicular lymphoma; DLBCL, diffuse large B-cell lymphoma; CLL, chronic lymphocytic leukemia; IV, intravenous; SC, subcutaneous; GemOx, gemcitabine and oxaliplatin; R, rituximab; B, bendamustine; CHOP, cyclophosphamide, doxorubicin, vincristine, prednisolone; DHAX/C, cytarabine, dexamethasone, and oxaliplatin/carboplatin.

### Glofitamab (RO7082859)

Glofitamab is a full-length BsAb with a 2:1 configuration with bivalency for CD20 on B cells and monovalency for CD3 on T cells. Results of the first-in-human phase I study have been published (29). R/R B-cell NHL patients were treated with single-agent in 14- or 21-day cycles (with obinutuzumab pretreatment 7 days before the first dose of glofitamab to reduce toxicity). One hundred seventy-one patients were treated, 74% with aggressive NHL. This trial included heavily pretreated patients, most refractory to prior therapy (90.6%) and with a median of 3 prior therapies. CRS occurred in 50.3% patients (grade 3 or 4: 3.5%); 1.2% experienced grade 3 ICAN. The ORR was 65.7% (CR, 57%) in those dosed at the recommended phase II dose. Of 63 patients with CR, 53 (84.1%) have ongoing CR with a maximum of 27.4 months observation. In patients with aggressive NHL treated with ≥ 10 mg, ORR was 61% with CR of 49%. Therefore, glofitamab demonstrated frequent and durable CRs with a manageable tolerability profile for patients with refractory B-NHL.

Glofitamab is also being tested in a number of combination trials for R/R and untreated B-cell NHL, such as in combination with chemotherapy as R-GemOx for R/R DLBCL or R-CHOP or R-CHP-polatuzumab vedotin in untreated DLBCL, in combination with monoclonal antibodies as atezolizumab or polatuzumab vedotin for R/R NHL, in combination with other BsAbs as the CD19 × CD3 RO7227166 for R/R NHL (**Table 2**).

### Epcoritamab (Gen3013)

Epcoritamab is a full-length IgG1 BsAb targeting CD3 and CD20 that is administered SC, leading to a gradual increase in drug levels and a lower peak in plasma cytokine levels. Results of the phase 1 trial have been also recently published (30). The primary objectives were to determine the maximum tolerated dose (MTD) and the recommended phase 2 dose. Sixty eight patients received escalating full doses (0·0128–60 mg) of epcoritamab, administered in 28-day cycles. No dose-limiting toxic effects were observed, and the MTD was not reached; the full dose of 48 mg was identified as the recommended phase 2 dose. Common adverse events were pyrexia 69%, CRS 59%, (all grade 1–2), and injection site reactions 47%. No discontinuations occurred due to treatment related adverse events. ORR was 68%, with 45% CR at full doses of 12–60 mg. Forty-eight patients had R/R DLBCL, with a median of 3 previous lines of therapy, 89% refractory to the previous line of therapy and 5 (11%) had relapsed to CART therapy. At 48 mg, ORR was 88%, with 38% CR. In conclusion, epcoritamab showed potent, single-agent, antitumor activity and an overall manageable safety profile, along with an easy way of administration.

Epcoritamab is currently being studied in two trials. The first one is a phase 1/2 study, in which epcoritamab is used in combination with other therapies in the relapsed or refractory setting as well as in previously untreated patients. Results of the first 9 patients treated with epcoritamab-R-CHOP have been presented. Preliminary data suggest that the combination has a manageable safety profile and all evaluable patients achieved early responses (31). It is also being evaluated in a phase 3 trial in patients with relapsed or refractory DLBCL (epcoritamab versus investigator choice of standard-of-care chemotherapy: R-GemOx or R-Bendamustine) (**Table 2**).

### Odronextamab (REGN1979)

Odronextamab is a fully human IgG4-based bispecific antibody targeting CD3 and CD20. Phase I data have been presented for R/R NHL (32). Odronextamab was administered using a step-up dose schedule consisting of an initial dose at week 1, an intermediate dose at week 2, and thereafter, a fixed weekly dose until week 12 followed by maintenance every other week dosing. One hundred twenty-seven patients with R/R B-NHL were treated at doses ranging from 0.03–320 mg (71 patients with DLBCL). Most patients were refractory to last therapy (80.3%) and had received a median of 3 (range: 1−11) prior lines of therapy; 29 (22.8%) patients had received prior CART therapy. No dose limiting toxicities were reported during dose escalation and MTD was not reached. The most frequent treatment-related adverse events were pyrexia (76.4%), CRS (62.2%), and chills (48.0%). Grade 3 CRS occurred in 8 (6.3%) patients and a grade 4 CRS occurred in 1 patient. Most of the CRS events occurred during the first 2 weeks of step-up dosing. Grade 3 neurologic events were noted in 5 (4.0%) patients; there were no grade 4 or higher neurologic adverse events. None of these events required treatment discontinuation. R/R DLBCL patients who had not received prior CART therapy, treated at doses ≥80 mg (n=10), ORR and CR rate were 60%; median observed DoR was 10.3 months (range 2.9–18.6+). In DLBCL patients who were refractory to prior CAR T therapy, treated at doses ≥80 mg (n=21), ORR was 33.3%, and CR rate was 23.8%; median observed DoR was 2.8 months (range 0 -18.9).

These results leaded to the pivotal phase 2 study currently enrolling for different disease groups, and a trial with the combination of odronextamab with the anti-PD-1 antibody cepilimab (**Table 2**).

## Discussion

Fortunately, new therapeutic strategies are available today to treat high risk R/R DLBCL patients. Three CD19 CART products, axi-cel, tisa-cel, and liso-cel, have been approved by FDA and EMA, to treat R/R DLBCL patients beyond 2 lines, and axi-cel has been recently approved by FDA as second line for adult patients with DLBCL refractory or relapsed within 12 months after first-line chemoimmunotherapy.

**Figure 1:**
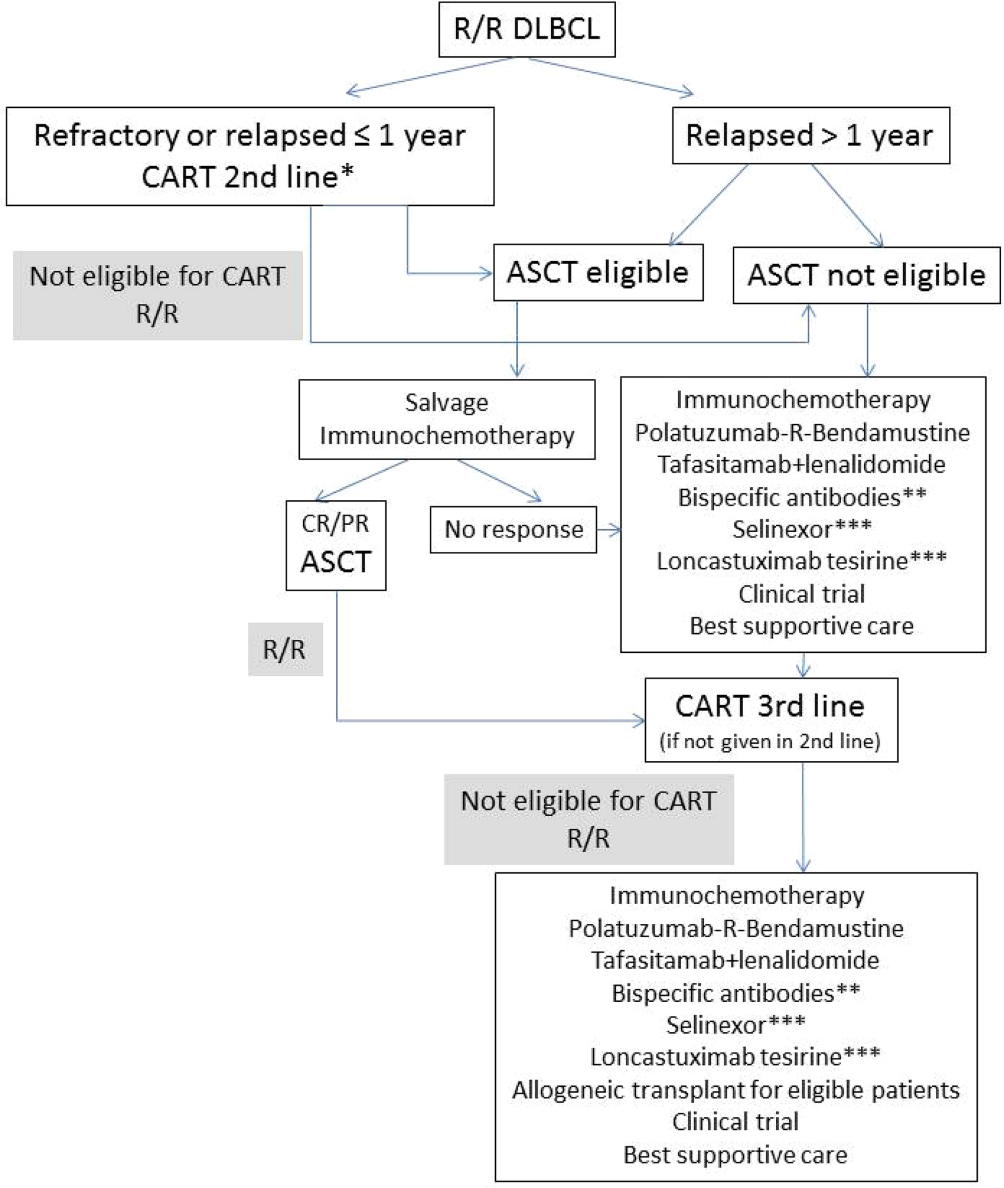
Proposed algorithm for the treatment of R/R DLBCL. R/R, relapsed or refractory; ASCT, autologous stem-cell transplant; CR, complete remission, PR, partial remission; *axi-cel approved by FDA; **currently available only in clinical trials; ***approved by FDA after ≥2 lines of previous therapy.

On the other hand, BsAbs have shown very promising preliminary results in the first published trials. Advantages and disadvantages of each of these strategies will be discussed, and a treatment algorithm is proposed for the treatment of R/R DLBCL (**Figure 1**).

CARTs have to be engineered for each individual patient, with a potential for logistical delays from the time of patient identification to CART infusion, as well as a risk of manufacturing failure (9–11). Moreover, CARTs may not be feasible for patients with rapidly progressive disease. These patients need bridging therapy during the manufacturing period, but it is not known what treatments are the best to be used. On the other hand, BsAbs, as an off-the-shelf option, allow immediate treatment. These agents are administered in an ease way, every 1 to 4 weeks either IV or even SC, and most can be administered in an outpatient basis. However, CART therapy consists in only one IV infusion, while treatment duration with BsAbs is prolonged.

Side effects associated with CARTs include CRS and ICANS, prolonged cytopenia, and impairment of humoral immunity with increased risk of infection (9–11). CRS is the most common and has been described in 42% to 93% of patients, with grade ≥3 events occurring in 2% to 22% of patients. BsAbs can also produce CRS and neurologic toxicities, seemingly at lower frequencies and severity, although toxicity data are not yet mature. Rates of CRS for BsAbs range from 27% (mosunetuzumab) to 62% (odronexamab), with grade ≥3 events in 0% (epcoritamab) to 7% (odronexamab) of patients (23, 29–31). Most of the CRS events occurred during the first 2 cycles. To mitigate CRS, different strategies are being employed, as the step-up dosing (23), the use SC formulation (24, 31), or the use a cytoreductive anti-CD20 monoclonal antibody before the BsAb infusion (29). Rates of ICANS of grade ≥3 range from 1% (glofitamab) to 4% (mosunetuzumab and odronexamab) with BsAbs. Additional toxicities described for BsAbs with an incidence of ≥10% include pyrexia, reaction at the injection site, and cytopenia. Like CARTs, BsAbs have been used safely in older patients and in patients with comorbidities (25).

Response rates for CARTs in patients with DLBCL range from 52% to 82%, with CR rates of 40% to 54%, and these responses are durable (9–12). For axi-cel, the CART product with a longer follow-up, with a median follow-up greater than 4 years, the OS at 4 years is 44% (12). For liso-cel, with a median follow-up of 40 months, the PFS at 3 years is 31%. For BsAbs, response rates in aggressive lymphomas range from 35% to 88%, with CRs of 19% to 60%, for those patients not exposed to prior CART. Nevertheless, follow-up with BsAbs is still very short, and most of the data regarding efficacy come from phase 1 trials. Therefore, efficacy data, although promising, are still immature, and the results of the new trials that are ongoing are needed to confirm them. Although CARTs offer durable responses, some patients will still relapse. BsAbs have been used in patients relapsed after CARTs, and durable responses have been observed (23, 31).

BsAbs have emerged as a novel class of off-the-shelf immunotherapies with a manageable safety profile, and clear efficacy in R/R aggressive B-cell lymphomas, including in those patients relapsing after CART therapy. There are still many open questions, and new trials are currently ongoing to confirm BsAbs efficacy and tolerability, as well as to explore its efficacy in different lines of therapy or in combination with other drugs.

## Author Contributions

EG performed the review, critically analyzed the data and wrote the manuscript.

## Funding

We thank CERCA Programme/Generalitat de Catalunya for institutional support.

## Conflict of Interest

EG declares having received lecture fees and advisory board fees from Janssen, Abbvie, Gilead, Kiowa, EUSAPharma, Incyte, Lilly, Beigene, Novartis, Abbvie, Takeda, and Roche.

## Publisher’s Note

All claims expressed in this article are solely those of the authors and do not necessarily represent those of their affiliated organizations, or those of the publisher, the editors and the reviewers. Any product that may be evaluated in this article, or claim that may be made by its manufacturer, is not guaranteed or endorsed by the publisher.
